# 1840. Implementation of a daptomycin dosing nomogram and assessment of clinical outcomes across a large health system

**DOI:** 10.1093/ofid/ofac492.1469

**Published:** 2022-12-15

**Authors:** Ariana Terravecchia, Vineet Gopinathan, Sarah B Minor

**Affiliations:** AdventHealth Orlando, Orlando, Florida; AdventHealth Orlando, Orlando, Florida; AdventHealth Orlando, Orlando, Florida

## Abstract

**Background:**

Daptomycin (DAP) is frequently used for a gram-positive infections with conflicting guidance on optimal dosing, especially dosing weight in obese patients. With newer commercially available vial sizes and limited supporting evidence pointing to an optimal dosing strategy and dosing weight (e.g., actual, ideal, adjusted), we seek to explore the impact of a standardized DAP nomogram (Figure 1) on both clinical and safety outcomes across a large health system.

**Figure 1.**
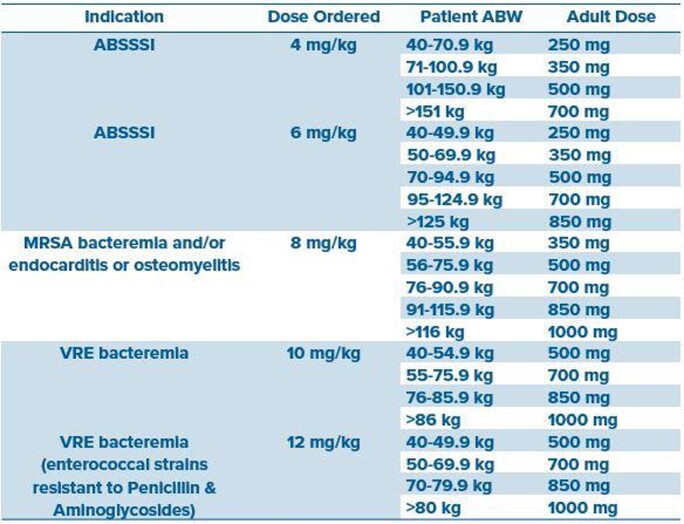
Standardized Daptomycin Dosing Nomogram

**Methods:**

This retrospective cohort study evaluated patients receiving DAP across 7 campuses within AdventHealth between Sep 1, 2020 to Dec 31, 2020 (pre-implementation with standard mg/kg dosing) and Dec 1, 2021 to Jan 31, 2022 (post-implementation with nomogram dosing). Inclusion criteria: age ≥18, invasive infection (e.g., bone/joint, bacteremia, complicated cellulitis, endocarditis), hospital admission, and duration of DAP ≥72hr. Exclusion: DAP in ambulatory setting, duration of DAP < 72hr, and non-invasive infection, defined as uncomplicated cellulitis not requiring surgical intervention. Primary outcome was clinical success, defined as improvement in signs/symptoms at end of therapy or discharge and absence of repeat positive cultures. Secondary outcomes included mean length of therapy (LOT), adherence to nomogram-guided DAP dosing, and occurrence of adverse events.

**Results:**

100 patients were included in each group. Average age was 58 years and most were male (54%). Most common indication for DAP was bacteremia (46%) and *S. aureu*s was isolated in 28% of all cultures. Thirty-four percent of patients in each group were on concomitant statin therapy and average BMI was similar between groups (31.4 kg/m^2^ vs. 31.9 kg.m2). Clinical success (Figure 2) occurred in 74% of patients in the post-implementation group compared to 70% in the pre-implementation group (p=0.529). Average LOT was similar (10.9 days vs. 9.15 days, p=0.191). Pharmacist adherence to the nomogram was high at 91%. Rates of adverse events were similar between groups, with CPK elevations > 200 units/L occurring in 12% of patients in the pre-implementation group compared to 14% of patients in the post-implementation group (p=0.674).

**Figure:**
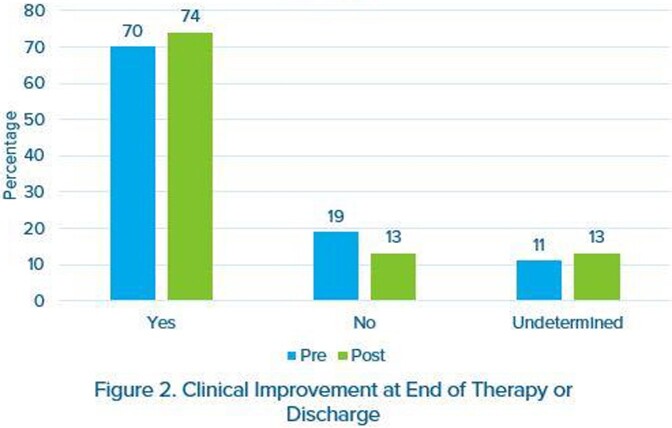

**Conclusion:**

A standardized DAP nomogram led to a safe and effective process for treating invasive gram-positive infections.

**Disclosures:**

**All Authors**: No reported disclosures.

